# The non-antibiotic macrolide EM900 attenuates HDM and poly(I:C)-induced airway inflammation with inhibition of macrophages in a mouse model

**DOI:** 10.1007/s00011-019-01302-3

**Published:** 2019-12-09

**Authors:** Hironori Sadamatsu, Koichiro Takahashi, Hiroki Tashiro, Go Kato, Yoshihiko Noguchi, Keigo Kurata, Satoshi Ōmura, Shinya Kimura, Toshiaki Sunazuka, Naoko Sueoka-Aragane

**Affiliations:** 1 Division of Haematology, Respiratory Medicine and Oncology, Department of Internal Medicine, Faculty of Medicine, Saga University, 5-1-1 Nabeshima, Saga, 849-8501 Japan; 2 Kitasato Institute for Life Sciences, Kitasato University, Tokyo, Japan; 3 Institute of Tokyo Environmental Allergy, Tokyo, Japan

**Keywords:** Asthma, EM900, HDM, Macrolide, Macrophage

## Abstract

**Objective:**

Macrolides have been reported to reduce the exacerbation of severe asthma. The aim of this study was to clarify the effects and mechanisms of EM900, a non-antibiotic macrolide, on allergic airway inflammation.

**Methods:**

Mice were sensitized and challenged by house dust mite (HDM), then exposed to polyinosinic-polycytidylic acid (poly(I:C)) as a model of asthma complicated with viral infection. Mice were administered with EM900. Airway inflammation was assessed from inflammatory cells in bronchoalveolar lavage fluid (BALF) and cytokines in lung tissues. Lung interstitial macrophages were counted by flow cytometry. Cytokine production, phosphorylation of NF-κB, and p38 in macrophages were examined by ELISA and western blotting.

**Results:**

Counts of cells in BALF and concentrations of IL-13, IL-5, RANTES, IL-17A, and MIP-2 were significantly decreased by EM900 compared to those without EM900. Percentages of lung interstitial macrophages were significantly decreased with EM900. Concentrations of IL-6, RANTES, and MIP-2 induced by HDM and poly(I:C) were significantly suppressed by EM900 through the suppression of NF-κB and p38 phosphorylation in macrophages.

**Conclusions:**

HDM and poly(I:C)-induced airway inflammation is attenuated by EM900 with the inhibition of lung interstitial macrophages. Clinical use of EM900 is expected, because EM900 has inhibitory effects against airway inflammation without inducing bacterial drug resistance.

**Electronic supplementary material:**

The online version of this article (10.1007/s00011-019-01302-3) contains supplementary material, which is available to authorized users.

## Introduction

Asthma is an airway inflammatory disease characterized by airway hyperresponsiveness (AHR) [1]. Eosinophilic airway inflammation is induced by sensitization and exposure to allergens such as house dust mite (HDM) [2]. Type 2 cytokines, interleukin (IL)-4, IL-5, and IL-13, are mainly involved in patients with asthma [3]. Inhaled corticosteroid (ICS) has contributed to disease control and reduction of mortality in recent decades [4]. However, exacerbation of asthma is frequently observed in patients with not only severe asthma, but also mild or controlled asthma. The most common cause of asthma exacerbation is respiratory infection, particularly viral infection.

Treatments for asthma have mainly been developed to address type 2 inflammation and eosinophilic inflammation. Recently, immunotherapies such as anti-immunoglobulin IgE, anti-IL-5, and anti-IL-4/IL-13 antibodies have become available for use in clinical situations [5–8]. However, molecular targeted therapies for non-type 2 inflammation have yet to be established. Macrolides that can be used clinically, such as erythromycin, clarithromycin (CAM) and azithromycin (AZM), have antibacterial effects, anti-inflammatory effects, and gastrointestinal motility-enhancing effects. In particular, anti-inflammatory effects have gained attention for the control of asthma. In some studies, macrolides have been reported to achieve anti-inflammatory effects and reduction of the exacerbation rate of asthma, especially with the use for non-type 2 airway inflammation. CAM contributes to improved asthma control through suppression of sputum IL-8 in non-eosinophilic severe asthma [9]. AZM decreased the frequency of asthma exacerbations among patients with adult asthma with persistent symptomatic asthma experience and improved quality of life, when administered for 48 weeks in both type 2 and non-type 2 asthma [10]. However, the detailed mechanisms by which macrolides improve asthma remain unclear and long-term use of macrolides for asthma is regarded as potentially problematic in terms of inducing antibiotic resistance in bacteria. EM900 is a 12-membered non-antibiotic macrolide derived from erythromycin and has been found to exert potent anti-inflammatory and immunomodulatory effects. EM900 has anti-inflammatory effects, but no antibacterial or gastrointestinal motility-enhancing effects. Given this absence of antibacterial effects, administration of EM900 carries no risk of inducing drug-resistant bacteria [11].

We have reported that monocytes recruited into the lungs represent cell sources of IL-33 in HDM-induced airway inflammation in mice [12]. Macrophage dysfunction is highly prevalent in asthma and macrophages are considered likely to play major roles in promoting the discrete inflammatory phenotypes of asthma [13].

The present study investigated the effects and mechanisms of EM900 in a mouse model of HDM and polyinosinic-polycytidylic acid (poly(I:C))-induced airway inflammation, including functions of macrophages.

## Materials and methods

### Allergens and chemicals

HDM extracts from *Dermatophagoides farinae* were purchased from ITEA (Tokyo, Japan). Poly(I:C) (Sigma-Aldrich, St. Louis, MO), as a synthetic analog of double-stranded (ds)RNA, was dissolved in phosphate-buffered saline (PBS). CAM (Tokyo Chemical Industry, Tokyo, Japan) was dissolved in dimethyl sulfoxide (DMSO) and diluted in PBS. Next, (8R,9S)-8,9-dihydro-6,9-epoxy-8,9-anhydropseudoerythromycin A (EM900), provided by Kitasato University, was dissolved in DMSO and diluted in PBS.

### Mice

Six-week-old female BALB/c mice (Japan SLC, Hamamatsu, Japan) were kept at the Saga University Animal Facility under specific pathogen-free conditions. Animal experiments were undertaken in accordance with the guidelines for the care and use of experimental animals by the Japanese Association for Laboratory Animals Science (1987) and were approved by the Saga University Animal Care and Use Committee.

### Protocol for airway inflammation in mice

Sensitization was achieved by intranasal administration of 25 μg HDM or PBS on days 1, 8, and 15. Exposure was carried out by intranasal administration of 10 μg HDM or PBS on days 22, 23, and 24. Mice were subsequently exposed by intranasal administration of 75 μg poly(I:C) or PBS on days 25 and 26 as the model of asthma complicated with viral infection. Mice were orally administered with placebo (PBS containing DMSO), 50 mg/kg CAM, or 25 mg/kg EM900 during exposure to poly(I:C) for 4 days (days 24, 25, 26, and 27). Placebo, CAM, or EM900 was administered after PBS or HDM administration on day 24, before 2 h of PBS or poly(I:C) administration on days 25 and 26 and before 2 h of collection of specimens on day 27. We used CAM, a representative macrolide, as a control to evaluate the anti-inflammatory effect of EM900. Finally, mice were divided into four groups: PBS-PBS-placebo (control group); HDM-poly(I:C)-placebo (HP group); HDM-poly(I:C)-CAM (CAM group); and HDM-poly(I:C)-EM900 (EM900 group). For all these models, mice were euthanized by intraperitoneal injection of midazolam, medetomidine, and butorphanol 24 h after the final poly(I:C) exposure on day 27. Bronchoalveolar lavage fluid (BALF) and lung tissues were collected for further analyses.

### Collection of BALF

BALF samples were collected as described previously [14]. Briefly, a 23-G tube was inserted into the trachea, followed by two lung lavages, each with 1 ml of saline. The cell suspension was centrifuged at 100×*g* for 5 min at 4 °C. The total number of cells was counted using a hemocytometer. Cytospin samples were prepared from the cell suspension. Cell differentiation was determined by counting at least 300 leukocytes in samples stained with Diff-Quik (Siemens, Munich, Germany).

### Histological examination of lung sections

Histological examinations were performed as previously reported [12]. Lungs were fixed in 10% neutral-buffered formalin (Wako, Osaka, Japan) and embedded in paraffin. Lung sections were stained with hematoxylin and eosin (HE) and periodic acid schiff (PAS). Slides were examined in a blinded fashion by three experienced observers, as previously described [15, 16]. For each slide, ten randomly chosen areas were scored. Peribronchial and perivascular inflammation was scored in a semiquantitative fashion on HE slides. Mucus deposition was scored in a semiquantitative fashion on PAS slides. Scoring was as follows: 0 = none; 1 = minimal; 2 = slight; 3 = moderate; and 4 = severe.

### Preparation of lung homogenates

After BAL, the right lung was isolated and homogenized in 50 mM Tris-buffered saline (pH 7.4) containing 1.0% Triton X-100, 0.1% sodium dodecyl sulfate, 150 mM sodium chloride, 0.5% sodium deoxycholate, 1 mM phenylmethylsulfonyl fluoride, 1 μg/ml aprotinin, 1 μg/ml leupeptin, and 1 mM Na_3_VO_4_. Lung homogenates were centrifuged at 10,000×*g* for 15 min, then supernatants were collected and stored at −80 °C until needed [14].

### AHR to methacholine

Mice were anesthetized with pentobarbital and xylazine before insertion into the exposed trachea of an 18-G metal needle connected to a flexiVent system (SCIREQ, Montreal, Canada) to apply the forced oscillation technique. Next, lungs were inflated to a pressure of 30 cmH_2_O, and baseline recordings were obtained using a single frequency (2.5 Hz, 1.2 s; Snapshot-150) and a broadband low frequency (1–20.5 Hz, 3 s; Quick-Prime-3). Mice were then exposed to an aerosol of saline. All parameters calculated from both test signals were recorded alternately every 10 s for 3 min. Finally, two deep lung inflations were administered. The above protocol was repeated five more times with aerosols containing sequentially increasing concentrations of 0.1, 1.0, 10, 20, and 50 mg/ml methacholine (Sigma-Aldrich Corp., St. Louis, IL).

### Isolation of single cells from lung tissue

Lung tissue was cut into small pieces then transferred through a 70-μm mesh before processing in a digestion buffer that included 0.02 mg/ml deoxyribonuclease I (Invitrogen, Waltham, MA) and 0.7 mg/ml collagenase type 2 (Worthington, Lakewood, NJ). The remaining red cells were lysed using BD Pharm Lysis (BD Biosciences, San Jose, CA) to obtain single-cell suspensions [12].

### Flow cytometry

Single-cell suspensions were pre-incubated with FcγR-specific blocking antibody and washed before staining. Cells were stained with CD45 (clone: 30-F11), CD11c (clone: N418), CD11b (clone: M1/70), and Ly6c (clone: HK1.4) (eBioscience, San Diego, CA) before collection on a flow cytometer (FACS Verse; BD Bioscience, Franklin Lakes, NJ) and analysis using FlowJo 8.3.3 software (Tree Star, Ashland, OR).

### Cell culture of MH-S and peritoneal macrophages (PEC)

The MH-S alveolar macrophage cell line was purchased from Public Health England (Porton Down, UK). MH-S was grown in RPMI 1640 medium containing 10% fetal calf serum. To obtain PEC, mice were injected intraperitoneally with 2 ml thioglycollate (3%) [17]. After 4 days, peritoneal fluid was obtained by lavage with 10 ml PBS. The fluid was centrifuged to isolate peritoneal macrophages, which were resuspended in RPMI 1640 medium. These cells were cultured at a density of 1 × 10^6^ cells in RPMI 1640 containing fetal calf serum and were stimulated. These macrophages were analyzed by enzyme-linked immunosorbent assay (ELISA) and western blotting.

### Quantification of cytokines using ELISA

IL-13, IL-5, regulated on activation, normal T cell expressed and secreted (RANTES), IL-17A, macrophage inflammatory protein 2 (MIP-2), IL-1β and monocyte chemoattractant protein-1 (MCP-1) were measured from lung homogenates using ELISA kits (R&D Systems, Minneapolis, MN), according to the instructions from the manufacturers. MH-S and PEC were stimulated with HDM and poly(I:C) and various concentrations of CAM or EM900 were added. After 24 h of stimulation, MCP-1, IL-6, RANTES, and MIP-2 were measured from culture supernatants using ELISA.

### Western blotting in MH-S and PEC

MH-S and PEC were stimulated with HDM and poly(I:C) and CAM or EM900 was added. After 60 min of stimulation, samples were washed with cold PBS buffer and lysed in lysis buffer containing 25% LDS sample buffer (NP0007; Invitrogen) and 5% DTT. Samples were boiled for 5 min, then loaded on sodium dodecyl sulfate polyacrylamide gel electrophoresis (SDS-PAGE) and transferred to nitrocellulose membranes (GE Healthcare Life Sciences, Chicago, IL). Membranes were blocked with 5% bovine serum albumin (Sigma-Aldrich Corp., St. Louis, IL). Membranes were incubated with the specific primary antibodies. After washing with Tris-buffered saline containing 0.1% Tween-20 (TBS-T), membranes were incubated with the secondary antibodies. After washing with TBS-T, membranes were incubated with ImmunoStar® LD containing luminescence solution or ImmunoStar® Zeta containing chemiluminescence solution (Wako). Films were scanned and protein bands were quantified using the C-DiGit® Blot Scanner (LI-COR). Antibodies of nuclear factor-κB (NF-κB) p65, phosphorylated NF-κB p65, p38, and phosphorylated p38 were purchased from Cell Signaling Technology (Danvers, MA) [18].

### Statistical analysis

Analysis of variance (ANOVA) was used for multiple comparisons of continuous variables. When a significant difference was identified, the difference between each group was tested using non-parametric Mann–Whitney *U* test. All tests were two-sided and significance was set at the level of *p* < 0.05. Data were analyzed using JMP Pro version 14 (SAS Institute Japan, Tokyo, Japan).

## Results

### EM900 suppressed HDM and poly(I:C)-induced airway inflammation

**Fig. 1:**
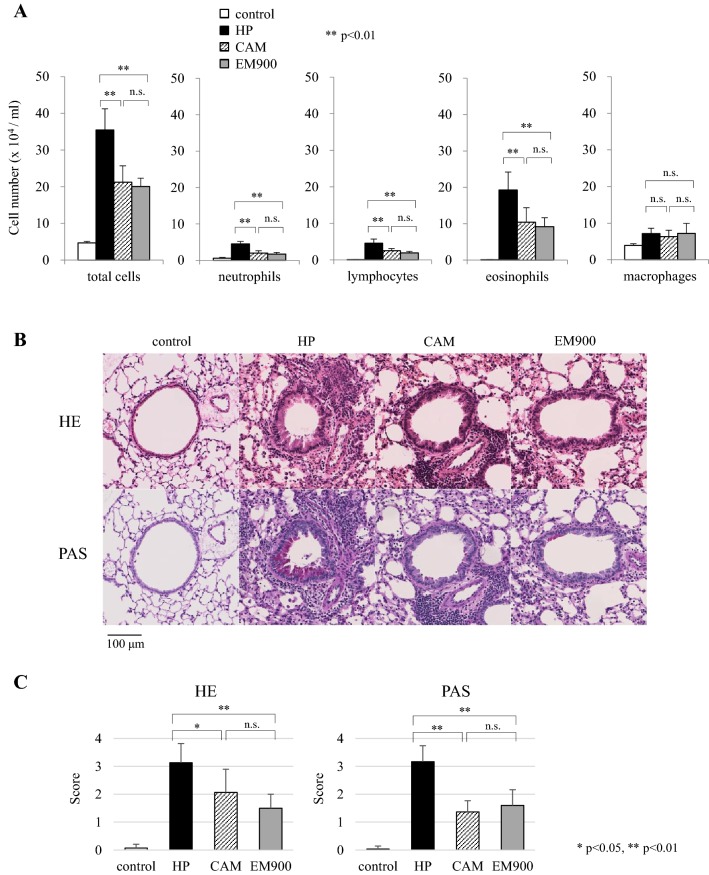

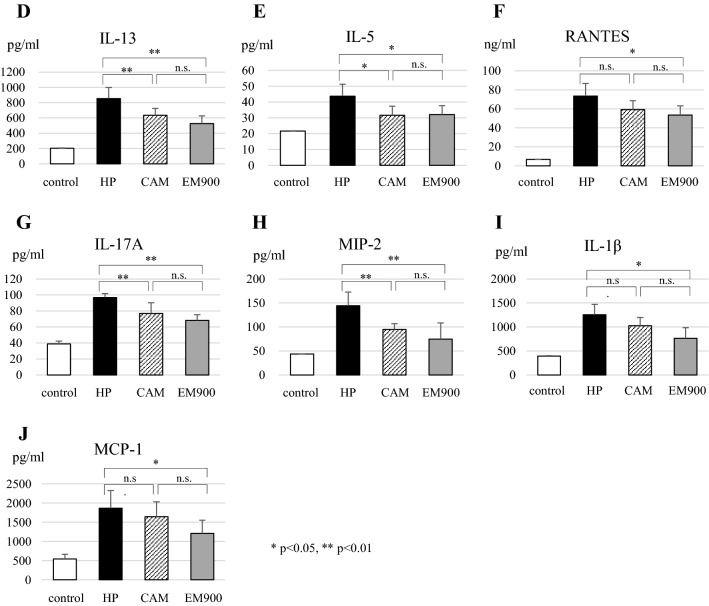
CAM or EM900 suppressed HDM and poly(I:C)-induced airway inflammation. **a** Bronchoalveolar lavage fluid analysis for total cell counts among control, HP, CAM, and EM900 mice (*n* = 6 in each group). **b** Histological examination for airway inflammation. Sections were stained with hematoxylin and eosin (HE) and periodic acid schiff (PAS). Original magnification, × 200. **c** Slides were scored for peribronchial inflammation and numbers of mucus-positive cells, using a semiquantitative score from 0 to 4. Histological scoring on HE and PAS stains is as follows: 0 = none; 1 = minimal; 2 = slight; 3 = moderate; or 4 = severe. Concentrations of IL-13 (D), IL-5 (E), RANTES (F), IL-17A (G), MIP-2 (H), IL-1β (I) and MCP-1 (J) in lung tissues were measured by ELISA (*n* = 6 in each group). Bar graphs represent mean ± standard deviation (SD) of four independent experiments. *p < 0.05, **p < 0.01, *n.s*. not significant, *CAM* clarithromycin, *HDM* house dust mite, *poly(I:C)* polyinosinic-polycytidylic acid, *RANTES* regulated on activation, normal T cell expressed and secreted, *MIP-2* macrophage inflammatory protein 2, *MCP-1* monocyte chemoattractant protein-1, *ELISA* enzyme-linked immunosorbent assay

We investigated the anti-inflammatory effects of CAM and EM900 in a mouse model of HDM and poly(I:C)-induced airway inflammation. Numbers of total cells, neutrophils, lymphocytes, and eosinophils in BALF were all significantly increased by HDM and poly(I:C) and were significantly suppressed by CAM or EM900 administrations. Numbers of macrophages were not suppressed by CAM or EM900 administrations. There was no significant difference between CAM and EM900 groups (Fig. 1a). On pathologic examination of the lungs, intense infiltration of inflammatory cell and marked mucus deposition were seen in the HP group and were significantly attenuated in the CAM and EM900 groups (Fig. 1b, c).

### EM900 suppressed both type 2 and non-type 2 airway inflammation

Levels of cytokines in lung tissues were measured by ELISA. Concentrations of IL-13, IL-5, RANTES, IL-17A, MIP-2, IL-1β, and MCP-1 were significantly increased by HDM and poly(I:C) administrations and were significantly suppressed by CAM or EM900 administrations, except for RANTES, IL-1β, and MCP-1 in the CAM group. There was no significant difference between CAM and EM900 groups (Fig. 1d–j). These results suggested that CAM and EM900 suppressed not only non-type 2 neutrophilic airway inflammation, but also type 2 eosinophilic airway inflammation.

In complementary studies, we investigated the anti-inflammatory effects of CAM and EM900 in a mouse model of HDM-induced airway inflammation without poly(I:C). Numbers of total cells, neutrophils, lymphocytes and eosinophils in BALF were not suppressed or increased by CAM or EM900 administrations (Supplementary Fig. 1a). Concentrations of cytokines in lung tissues were not suppressed by CAM or EM900 administrations, except for IL-5 in the EM900 (Supplementary Fig. 1b–f). Moreover, we investigated an effect of poly(I:C) in a mouse model of HDM-induced airway inflammation. Neutrophilic airway inflammation including numbers of neutrophils in BALF and concentrations of MIP-2 in lung tissues was significantly increased by addition of poly(I:C) compared with only HDM (Supplementary Fig. 2a–f).

### AHR is attenuated by EM900

**Fig. 2:**
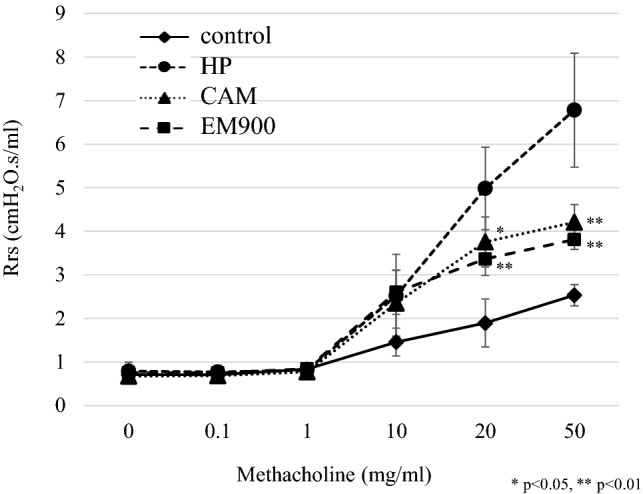
Airway hyperresponsiveness was attenuated by CAM or EM900. Airway hyperresponsiveness was measured through assessment of airway resistance according to graded concentrations of methacholine in control and HP, CAM, and EM900-treated mice (*n* = 6 in each group). Graphs represent mean ± standard deviation (SD) of four independent experiments. *p < 0.05, **p < 0.01 (compared with HP group). *CAM* clarithromycin

Airway resistance, represented by AHR, was measured by flexiVent. Airway resistance was higher in the HP group than in the control group and was significantly lower in the CAM and EM900 group mice than in the HP group after exposure to 20 and 50 mg/ml methacholine. There was no significant difference between CAM and EM900 groups (Fig. 2) (20 mg/ml methacholine; control: 1.90 ± 0.55 cmH_2_O·s/ml, HP: 4.98 ± 0.95 cmH_2_O·s/ml, CAM: 3.76 ± 0.57 cmH_2_O·s/ml, EM900: 3.36 ± 0.38 cmH_2_O·s/ml, 50 mg/ml methacholine; control: 2.53 ± 0.25 cmH_2_O·s/ml, HP: 6.78 ± 1.31 cmH_2_O·s/ml, CAM: 4.21 ± 0.41 cmH_2_O·s/ml, EM900: 3.81 ± 0.22 cmH_2_O·s/ml).

### Recruitment of interstitial macrophages into the lungs was attenuated by EM900

**Fig. 3:**
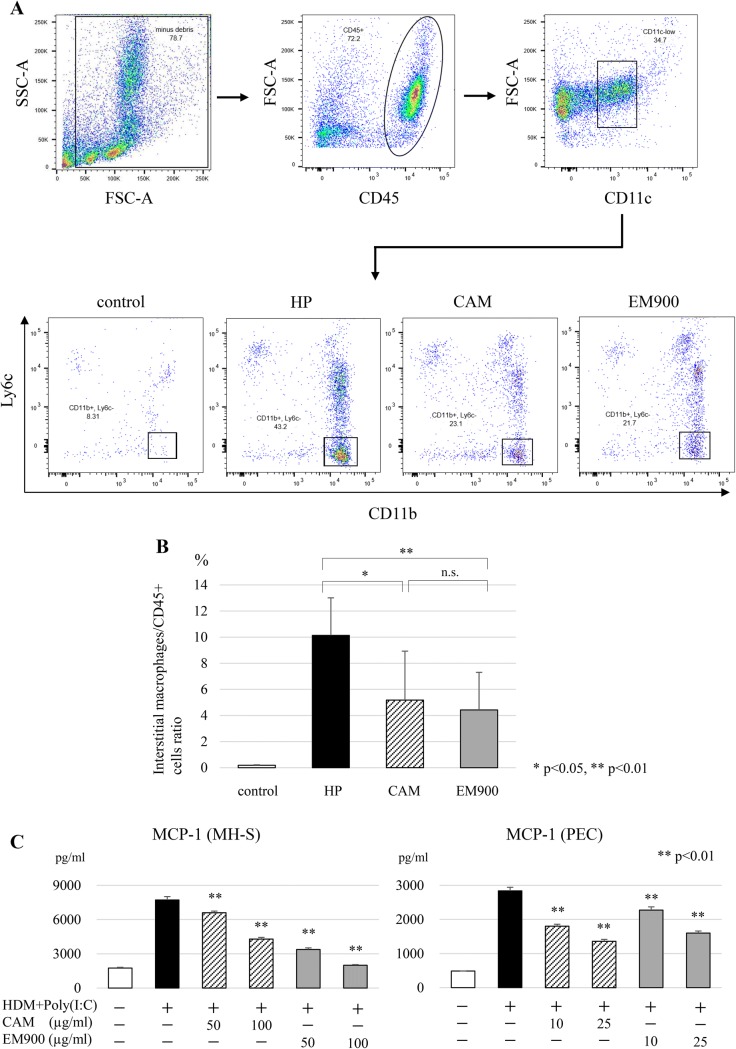
CAM or EM900 decreased numbers of lung macrophages. **a** Cells identified from digested lungs after exclusion of doublets and debris, leukocytes are separated by CD45 staining. CD11c-low, CD11b-positive, and Ly6c-negative cells are defined as interstitial macrophages. **b** Percentages of CD45^+^, CD11c^low^, CD11b^+^, and Ly6c^−^ cells in control and HP, CAM, and EM900-treated mice (*n* = 6 in each group). Bar graphs represent mean ± standard deviation (SD) of four independent experiments. *p < 0.05, **p < 0.01, *n.s*. not significant. **c** Concentration of MCP-1 in the supernatant of MH-S or PEC that are stimulated with HDM and poly(I:C) for 24 h. Bar graphs represent mean ± standard deviation (SD) of independent experiments. **p < 0.01 (compared with HDM + poly(I:C) without CAM and EM900). *CAM* clarithromycin, *MCP-1* monocyte chemoattractant protein-1, *PEC* peritoneal macrophages, *HDM* house dust mite, *poly(I:C)* polyinosinic-polycytidylic acid

To clarify the mechanisms underlying the effects of CAM and EM900 in HDM and poly(I:C)-induced airway inflammation, we focused on the action of macrophages. To identify lung cell populations in these mice, we examined single-cell suspensions by flow cytometry. Lung resident macrophages were classified as interstitial macrophages and alveolar macrophages [19]. We defined CD45^+^, CD11c^low^, CD11b^+^, and Ly6c^−^ cells as lung interstitial macrophages with reference to previous reports [20, 21]. The percentage of interstitial macrophages in the lungs was significantly increased by HDM and poly(I:C) administration and was significantly decreased by CAM or EM900 administrations. There was no significant difference between CAM and EM900 groups (Fig. 3a, b) (control 0.19 ± 0.04%; HP 10.14 ± 2.87%, CAM 5.18 ± 3.75%; EM900 4.43 ± 2.88%). Furthermore, we evaluated the production of MCP-1 from macrophages using MH-S and PEC in vitro. Concentrations of MCP-1 were significantly increased by HDM and poly(I:C) stimulations and were significantly suppressed by addition of CAM or EM900 in MH-S and PEC (Fig. 3c). These results (including Fig. 1j) suggested that CAM and EM900 attenuated the recruitment of interstitial macrophages in the lungs via suppression of MCP-1 production from macrophages.

### EM900 suppressed production of HDM and poly(I:C)-induced proinflammatory cytokines in vitro

**Fig. 4:**
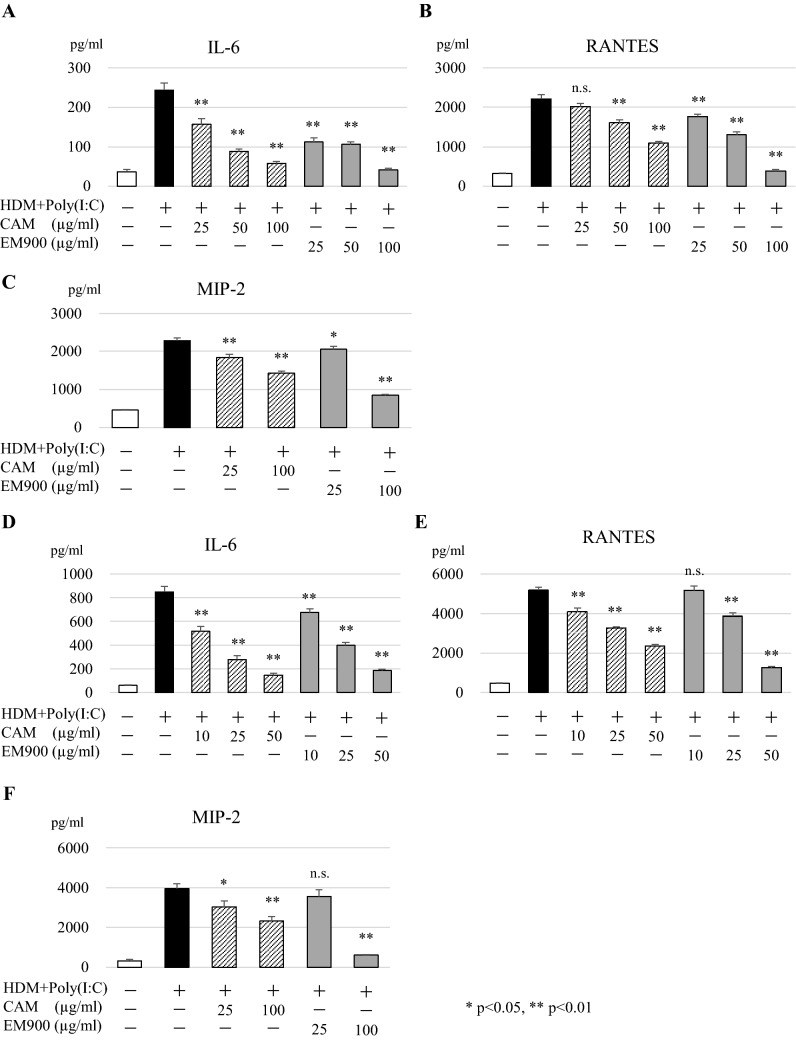
CAM or EM900 suppressed cytokine production in macrophages. Concentrations of IL-6 (**a**), RANTES (**b**), and MIP-2 (**c**) in supernatant from MH-S stimulated with HDM and poly(I:C) for 24 h. Concentrations of IL-6 (**d**), RANTES (**e**), and MIP-2 (**f**) of supernatant from PEC stimulated with HDM and poly(I:C) for 24 h. Bar graphs represent mean ± standard deviation (SD) of independent experiments. *p < 0.05, **p < 0.01, *n.s.* not significant, (compared with HDM + poly(I:C) without CAM and EM900). *CAM* clarithromycin, *RANTES* regulated on activation, normal T cell expressed and secreted, *MIP-2* macrophage inflammatory protein 2, *HDM* house dust mite, *poly(I:C)* polyinosinic-polycytidylic acid, *PEC* peritoneal macrophages

To clarify the interaction between macrolides and macrophages, we used in vitro assays of macrolides and macrophages. Concentrations of IL-6, RANTES, and MIP-2 were significantly increased by HDM and poly(I:C) stimulations and were significantly suppressed by CAM or EM900 in MH-S and PEC in a dose-dependent manner (Fig. 4a–f).

### EM900 attenuated NF-κB and p38 signaling in vitro

**Fig. 5:**
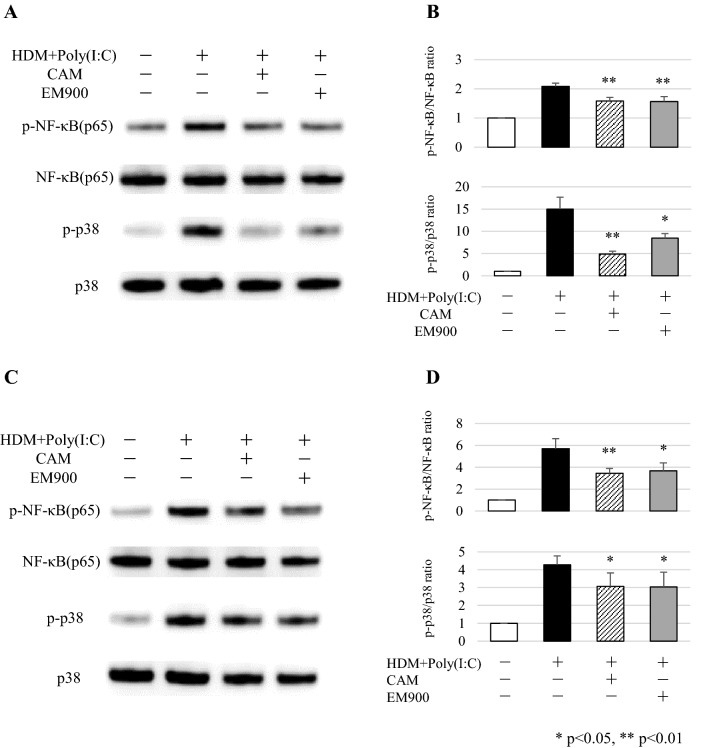
CAM or EM900 attenuated NF-κB and p38 signaling pathways in macrophages. **a** MH-S stimulated with 5 μg/ml HDM and 10 μg/ml poly(I:C) with or without 50 mg/ml CAM or 50 mg/ml EM900 for 60 min. After stimulation, cells were lysed and boiled for SDS-PAGE analysis. Transferred proteins were detected by anti-phosphorylated and total NF-κB (p65) or anti-phosphorylated and total p38 antibodies. **b** Protein bands quantified by densitometric analysis. **c** PEC stimulated with 5 μg/ml HDM and 10 μg/ml poly(I:C) with or without 50 mg/ml CAM or 50 mg/ml EM900 for 60 min. After stimulation, cells were lysed and boiled for SDS-PAGE analysis. Transferred proteins were detected by anti-phosphorylated and total NF-κB (p65) or anti-phosphorylated and total p38 antibodies. **d** Protein bands quantified by densitometric analysis. Bar graphs represent mean ± standard deviation (SD) of independent experiments. *p < 0.05, **p < 0.01 (compared with HDM + poly(I:C) without CAM and EM900). *CAM* clarithromycin, *HDM* house dust mite, *poly(I:C)* polyinosinic-polycytidylic acid, *PEC* peritoneal macrophages

To clarify the mechanisms by which macrolides act on macrophages, we investigated cell signaling pathways in MH-S and PEC by western blotting. Levels of phosphorylated NF-κB and phosphorylated p38-mitogen-activated protein kinase (MAPK) were significantly increased by HDM and poly(I:C) stimulations and were significantly suppressed by the addition of CAM or EM900 in MH-S and PEC (Fig. 5a–d). These results suggested that CAM and EM900 attenuated the production of proinflammatory cytokines from macrophages via the suppression of NF-κB and p38 signaling.

## Discussion

The present study demonstrated that the non-antibiotic macrolide EM900 attenuated HDM and poly(I:C)-induced airway inflammation with inhibition of macrophage recruitment and activation in a mouse model. EM900 decreased the number of eosinophils and neutrophils in BALF and inflammatory cells infiltration and mucus deposition in histological lung specimens and decreased production of cytokines, including IL-13, IL-5, RANTES, IL-17A, MIP-2, and IL-1β. IL-13, IL-5, and RANTES are mainly involved in type 2 eosinophilic airway inflammation and IL-17A, MIP-2, and IL-1β are mainly involved in non-type 2 neutrophilic airway inflammation. These results suggested that EM900 suppressed both type 2 and non-type 2 airway inflammation. To the best of our knowledge, this is the first report to demonstrate the effect of the non-antibiotic macrolide EM900 in HDM and poly(I:C)-induced airway inflammation in mice and to examine the mechanisms of action through lung interstitial macrophages.

Macrolides have been considered effective for anti-inflammatory effects and have reduced the exacerbation rate in severe asthma. The following findings have been shown in animal models. CAM attenuates airway inflammation via TNF*α *and IL-17A suppression in a mouse model of steroid-resistant asthma [22]. AZM attenuates ovalbumin (OVA)-induced airway inflammation in a mouse model [23]. AZM ameliorates OVA and lipopolysaccharide (LPS)-induced airway inflammation, both type 2 and non-type 2, in a mouse model [24]. The following findings have been shown in clinical studies. CAM contributes to improved asthma control through suppression of sputum IL-8 in non-eosinophilic severe asthma [9]. AZM decreased the frequency of asthma exacerbations in patients with adult asthma with persistent symptomatic asthma experience and improved quality of life [10]. This study showed that AZM is effective against both type 2 dominant and non-type 2 dominant asthma, but the mechanisms underlying these effects are not yet fully understood. The present study showed that EM900 or CAM suppressed both type 2 and non-type 2 inflammation. We consider that these results are because EM900 or CAM suppressed both RANTES and MIP-2, which are involved in eosinophil and neutrophil migration, respectively [25, 26].

We have shown that EM900 or CAM decreased not only the number of neutrophils, but also the number of eosinophils in an HDM and poly(I:C)-induced airway inflammation model. We considered three mechanisms by which EM900 or CAM could reduce eosinophils: reduction of RANTES; interaction between eosinophils and neutrophils; and relationship between macrophages and CD4 T-cells. First, the decline in eosinophils is attributed to decreased production of RANTES. RANTES is also known as CCL5, a C–C chemokine and has been reported as an eosinophil-attracting chemokine in airway inflammation. Eosinophil recruitment following allergen challenge is associated with the release of the chemokine RANTES into asthmatic airways [25]. In this study, RANTES were decreased by EM900 or CAM both in vivo and in vitro, which might be related to decreases in eosinophils. Second, inhibition of neutrophils may lead to inhibition of eosinophils. A previous study showed that the combination of neutrophils and LPS led eosinophils to accumulate in the airways [27]. In this study, neutrophils were suppressed by EM900 or CAM in BALF, which might be related to decreases in eosinophils. Finally, macrophages may be involved in CD4 T-cells through IL-6 production. Another study showed that alveolar macrophages from atopic asthmatics enhanced IL-5 production by allergen-specific CD4 T-cells due to their production of IL-6 [28]. EM900 could potentially have inhibited IL-5 through suppression of IL-6 production from macrophages and suppressed eosinophilic airway inflammation.

Long-term use of antibiotics is a problem in terms of inducing drug resistance in bacteria or mycobacteria [29]. Previous studies have shown that total antibiotic use correlates with penicillin-non-susceptible *Streptococcus pneumoniae* and macrolide-resistant *S. pneumoniae* in various countries [30]. Four years after treatment, high levels of the macrolide resistance gene *ermB* were still evident, indicating that antibiotic resistance, once selected for, can persist for longer than previously recognized [31]. Even with administration of CAM for a period of time as short as 7 days, resistant streptococci in the pharynx after 180 days were increased [32]. A few studies have shown the effects of non-antibiotic macrolides. GS-459755 and GS-560660 improved phagocytosis of macrophages in vitro and GS-459755 improved mucus clearance in human bronchial epithelial cells [33, 34]. EM900 is also a non-antibiotic macrolide that only shows anti-inflammatory effects [11]. A previous study showed that EM900 administration inhibited LPS-induced mucus production from rat nasal epithelium and inhibited the MUC5AC secretion induced by TNF*α* from human airway epithelial cells [35]. EM900 inhibits invasive pneumococcal infections by accelerating the clearance of pneumococcal nasopharyngeal colonization in mice [36]. EM900 is therefore considered to attenuate airway inflammation without inducing drug-resistant bacteria. The present report is the first to show the effects of the non-antibiotic macrolide for allergic airway inflammation.

The most common cause of asthma exacerbation is viral infection, at 76–80% [37]. Rhinovirus, respiratory syncytial virus, and influenza virus have been reported to be involved in asthma exacerbation [38, 39]. Genomic single-stranded RNA is converted to dsRNA in the infected cells. Virus-derived dsRNA induces inflammatory cytokine production in the respiratory tract of asthma patients and eosinophils and neutrophils accumulate in the respiratory tract, causing asthma exacerbation [40]. Several studies have used antigen (HDM or OVA) and poly(I:C) in virus-induced asthma exacerbation models in mice [41–44]. The present study therefore examined HDM and poly(I:C) administrations to mimic virus-induced asthma exacerbation. A previous study showed that EM900 was able to suppress IL-6 and IL-1β production in human tracheal epithelial cells after stimulation with rhinovirus RV14 [45]. EM900 induced a positive survival effect in influenza A virus infected mice [46]. These findings showed that EM900 can exert antiviral activity. EM900 also proved effective in the present study, and thus can be effective against virus-induced asthma exacerbation. Moreover, the finding that EM900 affects macrophages along with bronchial epithelial cells represents a novel insight.

We have previously shown mechanisms underlying the involvement of monocytes or macrophages in HDM-induced airway inflammation. IL-33 from monocytes recruited to the lung contributes to the pathogenesis of HDM-induced airway inflammation [12]. Saturated fatty acids increase the recruitment of lung macrophages and augments HDM-induced airway inflammation in obese mice [47]. In the present study, the number of interstitial macrophages (defined as CD45^+^, CD11c^low^, CD11b^+^, and Ly6c^−^ cells) was significantly decreased with EM900 or CAM treatment in HDM and poly(I:C)-induced airway inflammation in mice. Lung macrophages in asthma play roles in allergen recognition and production of inflammatory cytokines [48]. A recent study supports the notion that interstitial macrophages play a role in immune responses, including infection and allergic airway inflammation [20].

Cytokine concentrations of IL-6, RANTES, and MIP-2 induced by HDM and poly(I:C) were significantly suppressed by EM900 or CAM in vitro. Furthermore, activation of NF-κB and p38 was inhibited by EM900 or CAM treatment with HDM and poly(I:C) stimulation in macrophages. These data suggested that EM900 treatment directly affected macrophages through NF-κB and p38 in HDM and poly(I:C)-induced cytokine production. A previous study showed that p38-MAPK was associated with steroid resistance among patients with asthma [49]. Alveolar macrophages derived from asthmatic patients showed higher p38 activation in severe asthma than in mild asthma [49]. Production of IL-6 and IL-1β from alveolar macrophages was suppressed by administration of p38 inhibitor in combination with dexamethasone [50]. NF-κB activation is also reportedly associated with production of RANTES and IL-8 from peripheral blood mononuclear cells in patients with severe asthma [51]. NF-κB and p38 are involved in severe asthma or steroid resistance and control of these pathways is important.

In conclusion, HDM and poly(I:C)-induced airway inflammation is attenuated by EM900 via suppression of both type 2 and non-type 2 cytokines. Cytokine productions are suppressed by EM900 with inhibition of lung interstitial macrophages. Clinical use of EM900 is anticipated, because EM900 has inhibitory effects on airway inflammation equivalent to those of CAM and does not induce drug resistance to bacteria.

## Electronic supplementary material

Below is the link to the electronic supplementary material.

Supplementary file1 (PPTX 93 kb)
