# Supplementary material for: The non-antibiotic macrolide EM900 attenuates HDM and poly(I:C)-induced airway inflammation with inhibition of macrophages in a mouse model
Source: Inflamm Res. 2019 Dec 9;69(1):139–51. doi: 10.1007/s00011-019-01302-3 (PMC6942021; doi:10.1007/s00011-019-01302-3)
Supplement: Supplementary file 1 — Supplementary file1 (PPTX 93 kb) [file 11_2019_1302_MOESM1_ESM.pptx]

## Slide 1
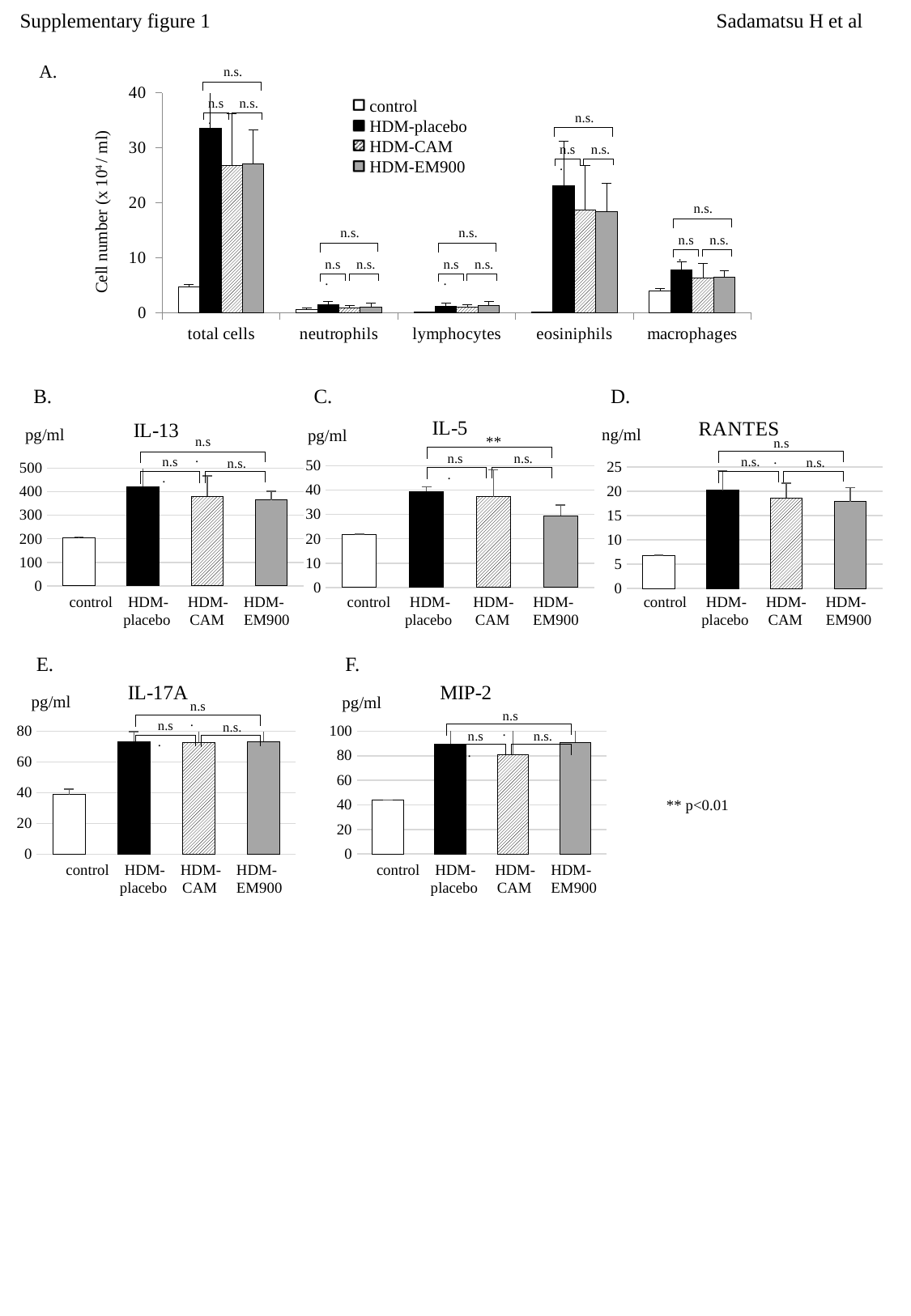

Supplementary figure 1
Sadamatsu H et al
A.
n.s.
n.s.
n.s.
### Chart
| Category | PBS-PBS-PBS | HDM-PBS-placebo | HDM-PBS-CAM | HDM-PBS-EM900 |
|---|---|---|---|---|
| | 4.676666666666667 | 33.53333333333333 | 26.783333333333335 | 27.099999999999998 |
| | 0.57756 | 1.4365166666666667 | 0.8076500000000001 | 1.0317 |
| | 0.08876333333333335 | 1.1921666666666666 | 0.9938833333333336 | 1.3023 |
| | 0.09086666666666667 | 23.092100000000002 | 18.699249999999996 | 18.369933333333332 |
| | 3.9194766666666667 | 7.812550000000002 | 6.2825500000000005 | 6.396066666666667 |control
HDM-placebo
HDM-CAM
HDM-EM900
n.s.
n.s.
n.s.
Cell number (x 104 / ml)
n.s.
n.s.
n.s.
n.s.
n.s.
n.s.
n.s.
n.s.
n.s.
C.
D.
B.
### Chart: IL-5
| Category | |
|---|---|
| control | 21.6266571963903 |
| HDM-PBS-placebo | 39.404761904761905 |
| HDM-PBS-CAM | 37.202380952380956 |
| HDM-PBS-EM900 | 29.46428571428571 |
### Chart: RANTES
| Category | |
|---|---|
| control | 6.666666666666667 |
| HDM-PBS-placebo | 20.157894736842103 |
| HDM-PBS-CAM | 18.578947368421055 |
| HDM-PBS-EM900 | 17.855263157894736 |
### Chart: IL-13
| Category | |
|---|---|
| control | 203.80952380952382 |
| HDM-placebo | 421.8333333333333 |
| HDM-CAM | 379.8333333333333 |
| HDM-EM900 | 365.0 |ng/ml
pg/ml
pg/ml
**
n.s.
n.s.
n.s.
n.s.
n.s.
n.s.
n.s.
n.s.
 control HDM- HDM- HDM-
 placebo CAM EM900
 control HDM- HDM- HDM-
 placebo CAM EM900
 control HDM- HDM- HDM-
 placebo CAM EM900
F.
E.
### Chart: MIP-2
| Category | |
|---|---|
| control | 43.693693693693696 |
| HDM-PBS-placebo | 89.27927927927927 |
| HDM-PBS-CAM | 81.03603603603602 |
| HDM-PBS-EM900 | 91.0810810810811 |
### Chart: IL-17A
| Category | |
|---|---|
| control | 38.71794871794872 |
| HDM-PBS-placebo | 73.33333333333333 |
| HDM-PBS-CAM | 72.73809523809523 |
| HDM-PBS-EM900 | 73.33333333333333 |pg/ml
pg/ml
n.s.
n.s.
n.s.
n.s.
n.s.
n.s.
** p<0.01
 control HDM- HDM- HDM-
 placebo CAM EM900
 control HDM- HDM- HDM-
 placebo CAM EM900

## Slide 2
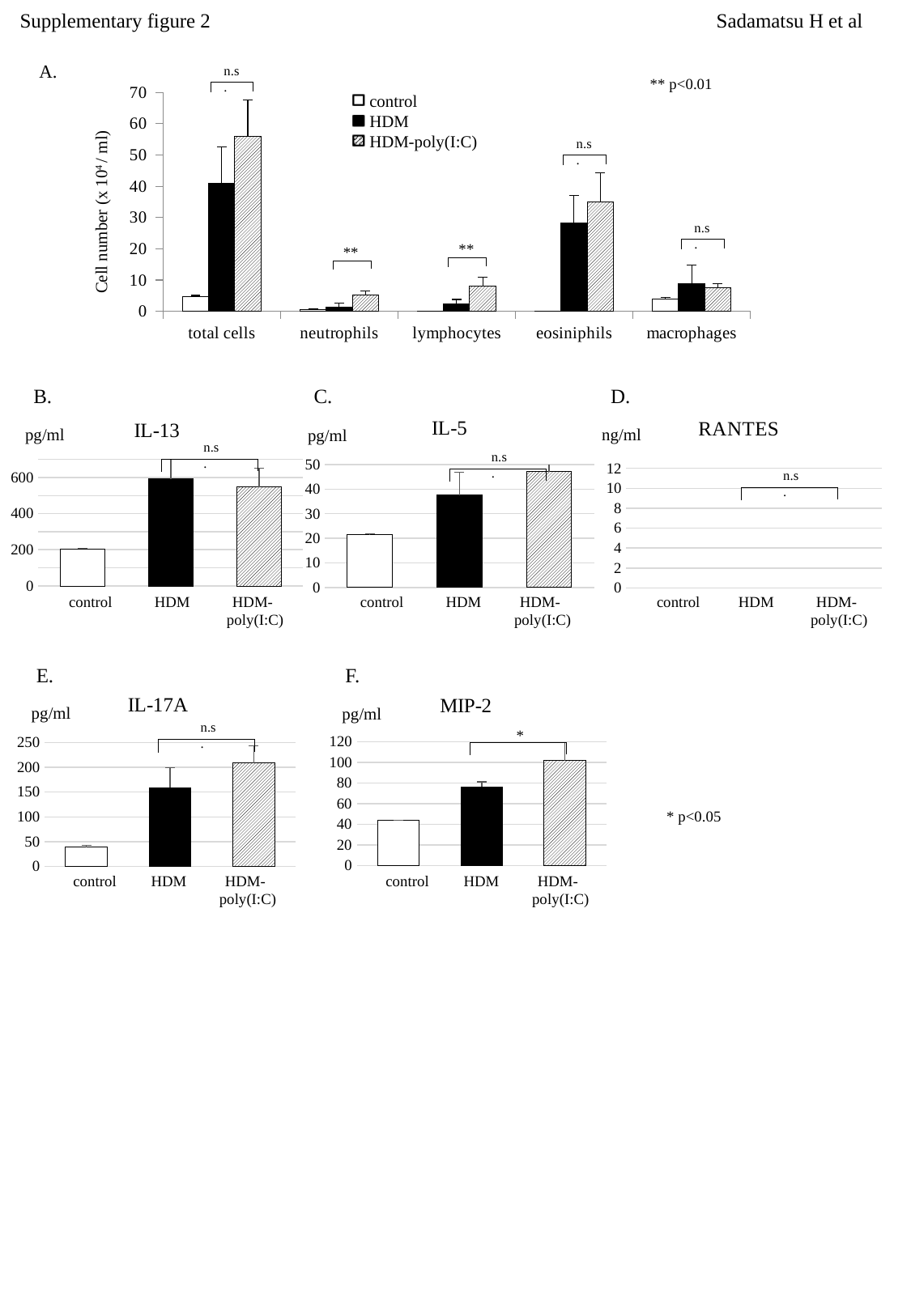

Supplementary figure 2
Sadamatsu H et al
A.
n.s.
** p<0.01
### Chart
| Category | control | HDM-PBS-placebo | HDM-Poly(I:C)-placebo |
|---|---|---|---|
| | 4.676666666666667 | 41.06666666666667 | 55.916666666666664 |
| | 0.57756 | 1.401441666666667 | 5.33585 |
| | 0.08876333333333335 | 2.4448416666666666 | 8.084733333333332 |
| | 0.09086666666666667 | 28.288700000000002 | 34.9876 |
| | 3.9194766666666667 | 8.931683333333332 | 7.508483333333333 |control
HDM
HDM-poly(I:C)
n.s.
Cell number (x 104 / ml)
n.s.
**
**
C.
D.
B.
### Chart: IL-5
| Category | |
|---|---|
| control | 21.6266571963903 |
| HDM-PBS | 37.6 |
| HDM-Poly(I:C) | 47.12 |
### Chart: IL-13
| Category | |
|---|---|
| control | 203.80952380952382 |
| HDM-PBS | 591.063492063492 |
| HDM-Poly(I:C) | 547.7777777777777 |
### Chart: RANTES
| Category | |
|---|---|
| control | 6.666666666666667 |
| HDM-PBS-placebo | 24.871794871794876 |
| HDM-Poly(I:C)-placebo | 31.91025641025641 |ng/ml
pg/ml
pg/ml
n.s.
n.s.
n.s.
 control HDM HDM-
 poly(I:C)
 control HDM HDM-
 poly(I:C)
 control HDM HDM-
 poly(I:C)
F.
E.
### Chart: MIP-2
| Category | |
|---|---|
| control | 43.693693693693696 |
| HDM-PBS | 76.26126126126125 |
| HDM-Poly(I:C) | 101.89189189189189 |
### Chart: IL-17A
| Category | |
|---|---|
| control | 38.71794871794872 |
| HDM-PBS | 158.55702917771887 |
| HDM-Poly(I:C) | 209.0291777188329 |pg/ml
pg/ml
n.s.
*
* p<0.05
 control HDM HDM-
 poly(I:C)
 control HDM HDM-
 poly(I:C)
